# Scientific Hypothesis-Testing Strengthens Neuroscience Research

**DOI:** 10.1523/ENEURO.0357-19.2020

**Published:** 2020-07-22

**Authors:** Bradley E. Alger

**Affiliations:** Department of Physiology and Program in Neuroscience, University of Maryland School of Medicine, Baltimore, MD, 21201

**Keywords:** estimation statistics, hypothesis testing, meta-analysis, p value, reproducibility crisis, statistical hypothesis

## Abstract

Science needs to understand the strength of its findings. This essay considers the evaluation of studies that test scientific (not statistical) hypotheses. A scientific hypothesis is a putative explanation for an observation or phenomenon; it makes (or “entails”) testable predictions that must be true if the hypothesis is true and that lead to its rejection if they are false. The question is, “how should we judge the strength of a hypothesis that passes a series of experimental tests?” This question is especially relevant in view of the “reproducibility crisis” that is the cause of great unease. Reproducibility is said to be a dire problem because major neuroscience conclusions supposedly rest entirely on the outcomes of single, *p* valued statistical tests. To investigate this concern, I propose to (1) ask whether neuroscience typically does base major conclusions on single tests; (2) discuss the advantages of testing multiple predictions to evaluate a hypothesis; and (3) review ways in which multiple outcomes can be combined to assess the overall strength of a project that tests multiple predictions of one hypothesis. I argue that scientific hypothesis testing in general, and combining the results of several experiments in particular, may justify placing greater confidence in multiple-testing procedures than in other ways of conducting science.

## Significance Statement

The statistical *p* value is commonly used to express the significance of research findings. But a single *p* value cannot meaningfully represent a study involving multiple tests of a given hypothesis. I report a survey that confirms that a large fraction of neuroscience work published in *The Journal of Neuroscience* does involve multiple-testing procedures. As readers, we normally evaluate the strength of a hypothesis-testing study by “combining,” in an ill-defined intellectual way, the outcomes of multiple experiments that test it. We assume that conclusions that are supported by the combination of multiple outcomes are likely to be stronger and more reliable than those that rest on single outcomes. Yet there is no standard, objective process for taking multiple outcomes into account when evaluating such studies. Here, I propose to adapt methods normally used in meta-analysis across studies to help rationalize this process. This approach offers many direct and indirect benefits for neuroscientists’ thinking habits and communication practices.

## Introduction

Scientists are not always clear about the reasoning that we use to conduct, communicate, and draw conclusions from our work, and this can have adverse consequences. A lack of clarity causes difficulties and wastes time in evaluating and weighing the strength of each others’ reports. I suggest that these problems have also influenced perceptions about the “reproducibility crisis” that science is reportedly suffering. Concern about the reliability of science has reached the highest levels of the NIH (Collins and Tabak, 2014) and numerous other forums (Landis et al., 2012; Task Force on Reproducibility, American Society for Cell Biology, 2014). Many of the concerns stem from portrayals of science like that offered by the statistician, John Ioannidis, who argues that “most published research findings are false,” especially in biomedical science (Ioannidis, 2005). He states “… that the high rate of nonreplication (lack of confirmation) of research discoveries is a consequence of the convenient, yet ill-founded strategy of *claiming conclusive research findings solely on the basis of a single study assessed by formal statistical significance, typically for a p value <0.05*” (italics added).

He continues, “Research is not most appropriately represented and summarized by *p* values, but, unfortunately, there is a widespread notion that medical research articles should be interpreted based only on *p* values.”

Additional concerns are added by Katherine Button and colleagues (Button et al., 2013), who conclude that much experimental science, such as neuroscience, is fatally flawed because its claims are based on statistical tests that are “underpowered,” largely because of small experimental group sizes. Statistical power is essentially the ability of a test to identify a real effect when it exists. Power is defined as “1-β,” where β is the probability of failing to reject the null hypothesis when it should be rejected. Statistical power varies from 0 to 1 and values of ≥0.8 are considered “good.” Button et al. (2013) calculate that the typical power of a neuroscience study is ∼0.2, i.e., quite low.

However, these serious concerns arise from broad assumptions that may not be universally applicable. Biomedical science encompasses many experimental approaches, and not all are equally susceptible to the criticisms. Projects in which multiple tests are performed to arrive at conclusions are expected to be more reliable than those in which one test is considered decisive. To the extent that basic (“pre-clinical”) biomedical science consists of scientific hypothesis testing, in which a given hypothesis is subjected to many tests of its predictions, it may be more reliable than other forms of research.

It is critical here to distinguish between a “scientific hypothesis” and a “statistical hypothesis,” which are very different concepts (Alger, 2019; chapter 5). A scientific hypothesis is a putative conceptual explanation for an observation or phenomenon; it makes predictions that could, in principle, falsify it. A statistical hypothesis is simply a mathematical procedure (often part of Null Hypothesis Significance Testing, NHST) that is conducted as part of a broader examination of a scientific hypothesis (Alger, 2019, p. 133). However, scientific hypotheses can be tested without using NHST methods, and, vice versa, NHST methods are often used to compare groups when no scientific hypothesis is being tested. Unless noted otherwise, in this essay “hypothesis” and “hypothesis testing” refer to scientific hypotheses.

To appreciate many of the arguments of Ioannidis, Button, and their colleagues, it is necessary to understand their concept of positive predictive value (PPV; see equation below). This is a statistical construct that is used to estimate the likelihood of reproducing a given result. PPV is defined as “the post-study probability that [the experimental result] is true” (Button et al., 2013). In addition to the “pre-study odds” of a result’s being correct, the PPV is heavily dependent on the *p* value of the result and the statistical power of the test. It follows from the statisticians’ assumptions about hypotheses and neuroscience practices that calculated PPVs for neuroscience research are low (Button et al., 2013). On the other hand, PPVs could be higher if their assumptions did not apply. I stress that I am not advocating for the use of the PPV, which can be criticized on technical grounds, but must refer to it to examine the statistical arguments that suggest deficiencies in neuroscience.

To look into the first assumption, that neuroscience typically bases many important conclusions on single *p* valued tests, I analyze papers published in consecutive issues of *The Journal of Neuroscience* during 2018. For the second assumption, I review elementary joint probability reasoning that indicates that the odds of obtaining a group of experimental outcomes by chance alone are generally extremely small. This notion is the foundation of the argument that conclusions derived from multiple experiments should be more secure those derived from one test. However, there is currently no standard way of objectively evaluating the significance of a collection of results. As a step in this direction, I use two very different procedures, Fisher’s method of combining results and meta-analysis of effect sizes (Cummings and Calin-Jageman, 2017) measured by Cohen’s *d*, which have not, as far as I know, been applied to the problem of combining outcomes in the way that we need. Finally, in Discussion, I suggest ways in which combining methods such as these can improve how we assess and communicate scientific findings.

## Materials and Methods

To gauge the applicability of the statistical criticisms to typical neuroscience research, I classified all Research Articles that appeared in the first three issues of *The Journal of Neuroscience* in 2018 according my interpretation of the scientific “modes” they represented, i.e., “hypothesis testing,” “questioning,” etc., because these modes have different standards for acceptable evidence. Because my focus is on hypothesis testing, I did a pdf search of each article for “hypoth” (excluding references to “statistical” hypothesis and cases where “hypothesis” was used incorrectly as a synonym for “prediction”). I also searched “predict” and “model” (which was counted when used as a synonym for “hypothesis” and excluded when it referred to “animal models,” “model systems,” etc.) and checked the contexts in which the words appeared. In judging how to categorize a paper, I read its Abstract, Significance Statement, and as much of the text, figure legends, and Discussion as necessary to understand its aims and see how its conclusions were reached. Each paper was classified as “hypothesis-based,” “discovery science,” (identifying and characterizing the elements of an area), “questioning” (a series of related questions not evidently focused on a hypothesis), or “computational-modeling” (where the major focus was on a computer model, and empirical issues were secondary).

I looked not only at what the authors said about their investigation, i.e., whether they stated directly that they were testing a hypothesis or not, but what they actually did. As a general observation, scientific authors are inconsistent in their use of “hypothesis,” and they often omit the word even when it is obvious that they are testing a hypothesis. When the authors assumed that a phenomenon had a specific explanation, then conducted experimental tests of logical predictions of that explanation, and drew a final conclusion related to the likely validity of the original explanation, I counted it as implicitly based on a hypothesis even if the words “hypothesis,” “prediction,” etc. never appeared. For all hypothesis-testing papers, I counted the number of experimental manipulations that tested the main hypothesis, even if there were one or more subsidiary hypotheses (see example in text). If a paper did not actually test predictions of a potential explanation, then I categorized it as “questioning” or “discovery” science. While my strategy was unavoidably subjective, the majority of classifications would probably be uncontroversial and disagreements unlikely to change the overall trends substantially.

To illustrate use of the statistical combining methods, I analyzed the paper by Cen et al. (2018), as suggested by a reviewer of the present article. The authors made multiple comparisons with ANOVAs followed by Bonferroni *post hoc* tests; however, to make my analysis more transparent, I measured means and SEMs from their figures and conducted two-tailed *t* tests. When more than one experimental group was compared with the same standard control, I took only the first measurement to avoid possible complications of non-independent *p* values. I used the *p* values to calculate the combined mean significance level for all of the tests according to Fisher’s method (see below). This is an extremely conservative approach, as including the additional tests would have further increased the significance of the combined test.

For the meta-analysis of the Cohen’s *d* parameter (Cummings and Calin-Jageman, 2017; p. 239), I calculated effect sizes on the same means and SEMs from which *p* values were obtained for the Fisher’s method example. I determined Cohen’s *d* using an on-line calculator (https://www.socscistatistics.com/effectsize/default3.aspx) and estimated statistical power with G*-Power (http://www.psychologie.hhu.de/arbeitsgruppen/allgemeine-psychologie-und-arbeitspsychologie/gpower.html). I then conducted a random-effects meta-analysis on the Cohen’s *d* values with Exploratory Software for Confidence Interval (ESCI) software, which is available at https://thenewstatistics.com/itns/esci/ (Cummings and Calin-Jageman, 2017).

## Results

Of the total of 52 Research Articles in the first three issues of *The Journal of Neuroscience* in 2018, I classified 39 (75%) as hypothesis-based, with 19 “explicitly” and 20 “implicitly” testing one or more hypotheses. Of the remaining 13 papers, eight appeared to be “question” or “discovery” based, and five were primarily computer-modeling studies that included a few experiments (see Table 1). Because the premises and goals of the non-hypothesis testing kinds of studies are fundamentally distinct from hypothesis-testing studies (Alger, 2019; chp. 4), the same standards cannot be used to evaluate them, and I did not examine these papers further.

None of the papers based its major conclusion on a single test. In fact, the overarching conclusion of each hypothesis-based investigation was supported by approximately seven experiments (6.9 ± 1.57, mean ± SD, *n* = 39) that tested multiple predictions of the central hypothesis. In 20 papers, at least one (one to three) alternative hypothesis was directly mentioned. Typically (27/39), the experimental tests were “consistent” with the overall hypothesis, while in 19 papers, at least one hypothesis was explicitly falsified or ruled out. These results replicate previous findings (Alger, 2019; chapter 9).

As noted earlier, some science criticism rests on the concept that major scientific conclusions rest on the outcome of a single *p* valued test. Indeed, there are circumstances in which the outcome of a single test is intended to be decisive, for instance, in clinical trials of drugs where we need to know whether the drugs are safe and effective or not. Nevertheless, as the preceding analysis showed, the research published in *The Journal of Neuroscience* is not primarily of this kind. Moreover, we intuitively expect conclusions bolstered by several lines of evidence to be more secure than those resting on just one. Simple statistical principles quantify this intuition.

Provided that individual events are truly independent—the occurrence of one does not affect the occurrence of the other and the events are not correlated—then the rule is to multiply their probabilities to get the probability of the joint, or compound, event in which all of the individual events occur together or sequentially. Consider five games of chance with probabilities of winning of 1/5, 1/15, 1/20, 1/6, and 1/10. While the odds of winning any single game are not very small, if you saw someone step up and win all five in a row, you might well suspect that he was a cheat, because the odds of doing that are 1/90,000.

The same general reasoning applies to the case in which several independent experimental predictions of a given hypothesis are tested. If the hypothesis is that GABA is the neurotransmitter at a given synapse, then we could use different groups of animals, experimental preparations, etc. and test five independent predictions: that synaptic stimulation will evoke an IPSP; chemically distinct pharmacological agents will mimic and block the IPSP; immunostaining for the GABA-synthetic enzyme will be found in the pre-synaptic nerve terminal; the IPSP will not occur in a GABA receptor knock-out animal, etc. The experiments test completely independent predictions of the same hypothesis, hence the chance probability of obtaining five significant outcomes that are consistent with it by random chance alone must be much lower than that of obtaining any one of them. If the tests were done at *p* ≤ 0.05, the chances would be ≤(0.05)^5^ or ≤3.13^−7^ that they would all just happen to be consistent with the hypothesis. Accordingly, we feel that a hypothesis that has passed many tests to be on much firmer ground than if it had passed only one test. Note, however, that the product of a group of *p* values is just a number; it is not itself a significance level.

It can be difficult for readers to tease the crucial information out of scientific papers as they are currently written. Not only is the work intrinsically complicated, but papers are often not written to maximize clarity. A common obstacle to good communication is the tendency of scientific papers to omit a direct statement of the hypotheses that are being tested, which is an acute problem in papers overflowing with data and significance tests. An ancillary objective of my proposal for analysis is to encourage authors to be more straightforward in laying out the logic of their work. It may be instructive to see how a complex paper can be analyzed.

As an example, I used the paper of Cen et al. (2018). Although the paper reports a total of 114 *p* values, they do not all factor equally in the analysis. The first step is to see how the experiments are organized. The authors state that their main hypothesis is that N-cadherin, regulated by PKD1, promotes functional synapse formation in the rodent brain. It appears that the data in the first two figures of the paper provide the critical tests of this hypothesis. These figures include 43 statistical comparisons, many of which were controls to ensure measurement validity, or which did not critically test the hypothesis, e.g., 18 tests of spine area or miniature synaptic amplitude were supportive, but not critical. I omitted them, as well as multiple comparisons made to the same control group to avoid the possibility of correlations among *p* values. For instance, if an effect was increased by PKD1 overexpression (OE) and reduced by dominant negative (DN) PKD1, I counted only the increase, as both tests used the same vector-treated control group. In the end, six unique comparisons tested crucial, independent, non-redundant predictions (shown in Figs. 2*A2*,*B2*,*D2*,*E2*, 3*B2*,*C2* of Cen et al., 2018). I emphasize that this exercise is merely intended to illustrate the combining methods; the ultimate aim is to encourage authors to explain and justify their decisions about including or excluding certain tests in their analyses.

Cen et al. (2018) test the following predictions of their main hypothesis with a variety of morphologic and electrophysiological methods:

(1) N-cadherin directly interacts with PKD1.Test: GST pull-down.

(2) N-cadherin and PKD1 will co-localize to the synaptic region. Test: immunofluorescence images.

Predictions (1) and (2) are descriptive, i.e., non-quantified; other predictions are tested quantitatively.

(3) PKD1 increases synapse formation. Tests (Fig. 2*A2*): OE of hPKD1 increases spine density and area (*p* < 0.001 for both); DN-hPKD1 decreases spine density and area (*p* < 0.001 for both).

(4) PKD1 increases synaptic transmission. Tests (Fig. 2*B2*): OE of hPKD1 increases mEPSC frequency (*p* < 0.006) but not amplitude; DN-hPKD1 decreases mEPSC frequency (*p* < 0.002) but not amplitude.

(5) PKD1 acts upstream of N-cadherin on synapse formation and synaptic transmission. Tests (Fig. 3*B2*): DN-hPKD-induced reductions of spine density and area are rescued by OE of N-cadherin (*p* < 0.001 for both). DN-hPKD1-induced reduction in mEPSC frequency is rescued by OE of N-cadherin (*p* < 0.001 for both).

**Figure 1.**
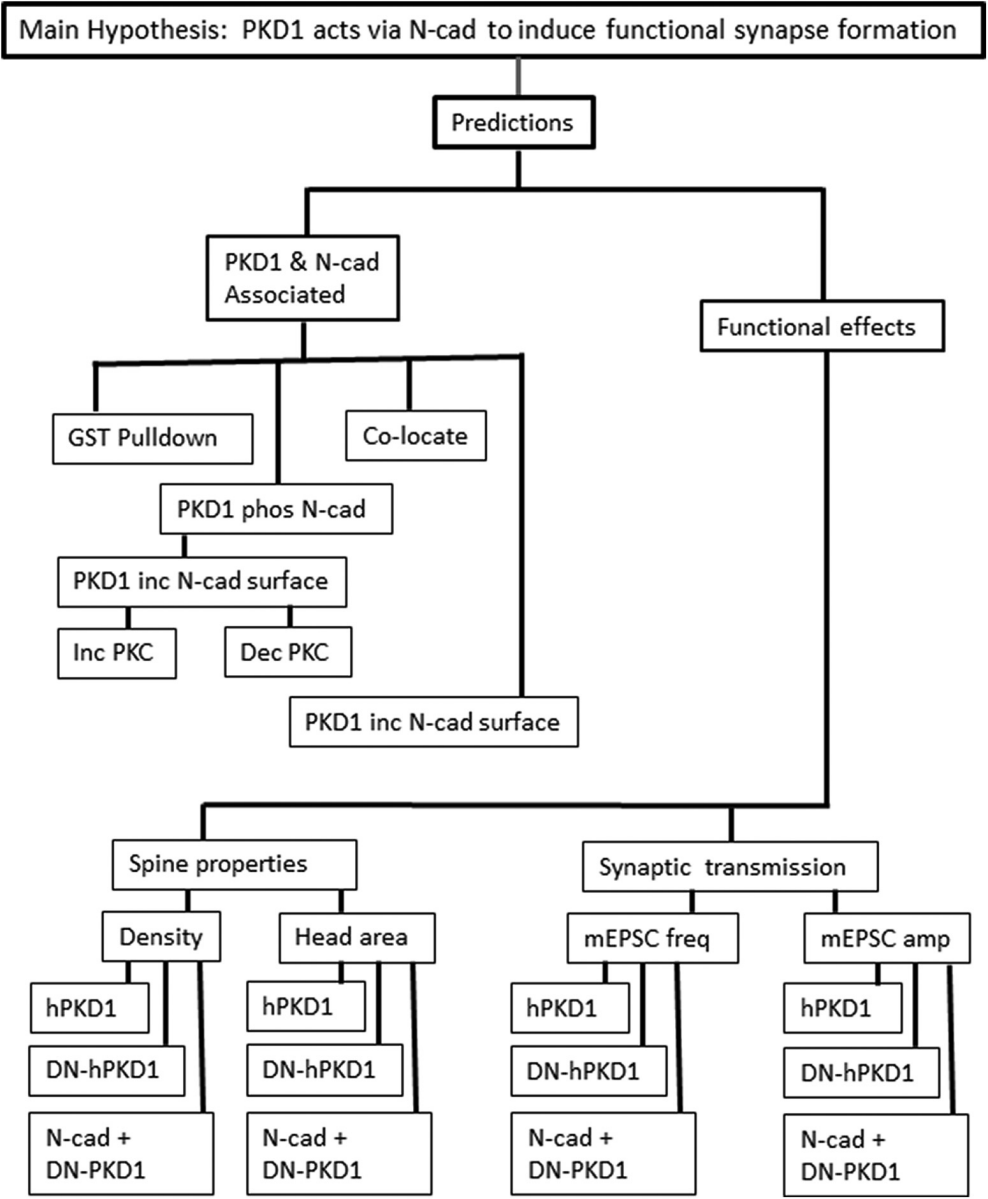
Diagram of the main hypothesis and predictions of Cen et al. (2018). The solid lines connect the hypothesis and the logical predictions tested. This diagram omits experimental controls tests that primarily validate techniques, include non-independent *p* values, or add useful but non-essential information. The main hypothesis predicts that PKD1 associates directly with N-cadherin, and that PKD1 and N-cadherin jointly affect synaptic development in a variety of structural and physiological ways. Separate groups of experiments test these predictions.

These are the key predictions the main hypothesis: their falsification would have called for the rejection of the hypothesis in its present form. The organization of these tests of the main hypothesis is illustrated in Figure 1.

**Figure 2.**
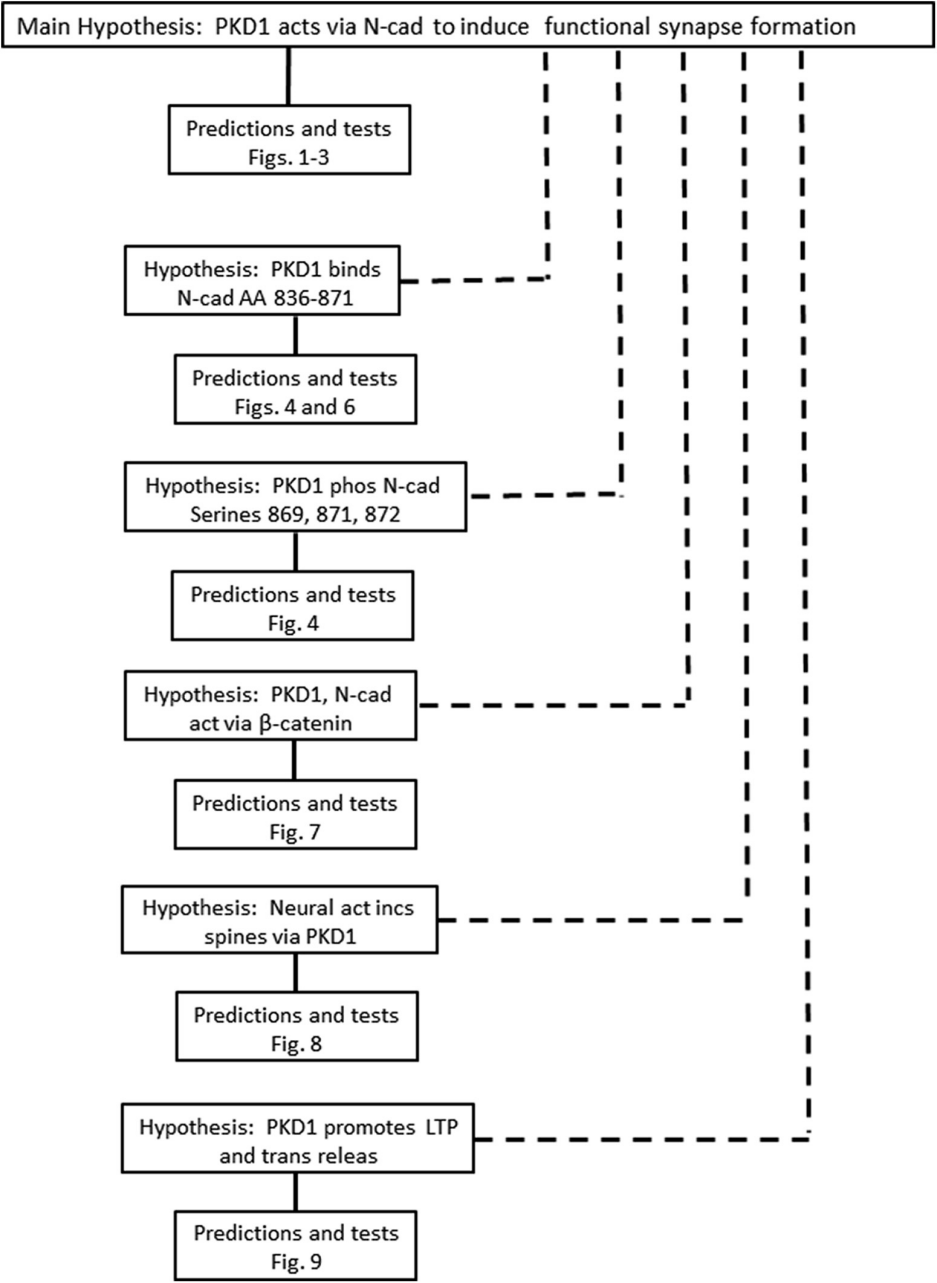
Diagram of the logical structure of Cen et al. (2018). The paper reports several distinct groups of experiments. One group tests the main hypothesis and others test subsidiary hypotheses that are complementary to the main one but are not a necessary part of it. Connections between hypotheses and predictions that are logically necessary are indicated by solid lines; dotted lines indicate complementary, but not mandatory, connections. Falsification of the logically-necessary predictions would call for rejection of the hypothesis in its present form; falsification of any of the subsidiary hypothesis would not affect the truth of the main hypothesis. The figure numbers in the boxes identify the source of major data in Cen et al., 2018 that were used to test the indicated hypothesis.

Cen et al. (2018) go on to identify specific sites on N-cadherin that PKD1 binds and phosphorylates and they test the associated hypothesis that these sites are critical for the actions of PKD1 on N-cadherin. They next investigate β-catenin as a binding partner for N-cadherin and test the hypothesis that this binding is promoted by PKD1. While these subsidiary hypotheses and their tests clearly complement and extend the main hypothesis, they are distinct from it and must be analyzed separately. Whether falsified or supported, the outcomes of testing them would not affect the conclusion of the main hypothesis. The relationships among the main hypothesis and other hypotheses are shown in Figure 2. Note that Cen et al. (2018) is unusually intricate, although not unique; the diagrams of most papers will not be nearly as complicated as Figures 1, 2.

Basic probability considerations imply that the odds of getting significant values for all six critical tests in Cen et al. (2018) by chance alone are extremely tiny; however, as mentioned, the product of a group of *p* values is not a significance level. R.A. Fisher introduced a method for converting a group of independent *p* values that all test a given hypothesis into a single parameter that can be used in a significance test (Fisher, 1925; Winkler et al., 2016; see also Fisher’s combined probability test https://en.wikipedia.org/wiki/Fisher’s_method; https://en.wikipedia.org/wiki/Extensions_of_Fisher’s_method). For convenience, I will call this parameter “p_FM_” because it is not a conventional *p* value. Fisher’s combined test is used in meta-analyses of multiple replications of the same experiment across a variety of conditions or laboratories but has not, to my knowledge, been used to evaluate a collection of tests of a single scientific hypothesis. Fisher’s test is:

$$\chi^{2}=-2\sum_{i=1}^{k}\ln\;(p_{i}),$$

where *p*_*i*_ is the *p* value of the i^th^ test and there are *k* tests in all. The sum of the natural logarithms (*ln*) of the *p* values, multiplied by −2, is a χ^2^ variable with 2*k* degrees of freedom and can be evaluated via a table of critical values for the χ^2^ distribution (for derivation of Fisher’s test equation, see: https://brainder.org/2012/05/11/the-logic-of-the-fisher-method-to-combine-p-values/). Applying Fisher’s test to Cen et al.’s major hypothesis (k = 6; df = 12), yields

$$X^{2}\;=\;35.95\;\text{or}\;p<0.001.$$

In other words, the probability, p_FM_, of getting their collection of *p* values by chance alone is <0.001, and therefore, we may be justified having confidence in the conclusion. Fisher’s method, or a similar test, does not add any new element but gives an objective estimate of the significance of the combined results. (Note here that the mathematical transformation involved can yield results that differ qualitatively from simply multiplying the *p* values.) I stress that Fisher’s method is markedly affected by any but the most minor correlations (i.e., *r* > 0.1) among *p* values; notable correlations among these values will cause p_FM_ to be much lower (i.e., more extreme) than the actual significance value (Alves and Yu, 2014; Poole et al., 2016). Careful experimental design is required to ensure the independence of the tests to be combined.

**Figure 3.**
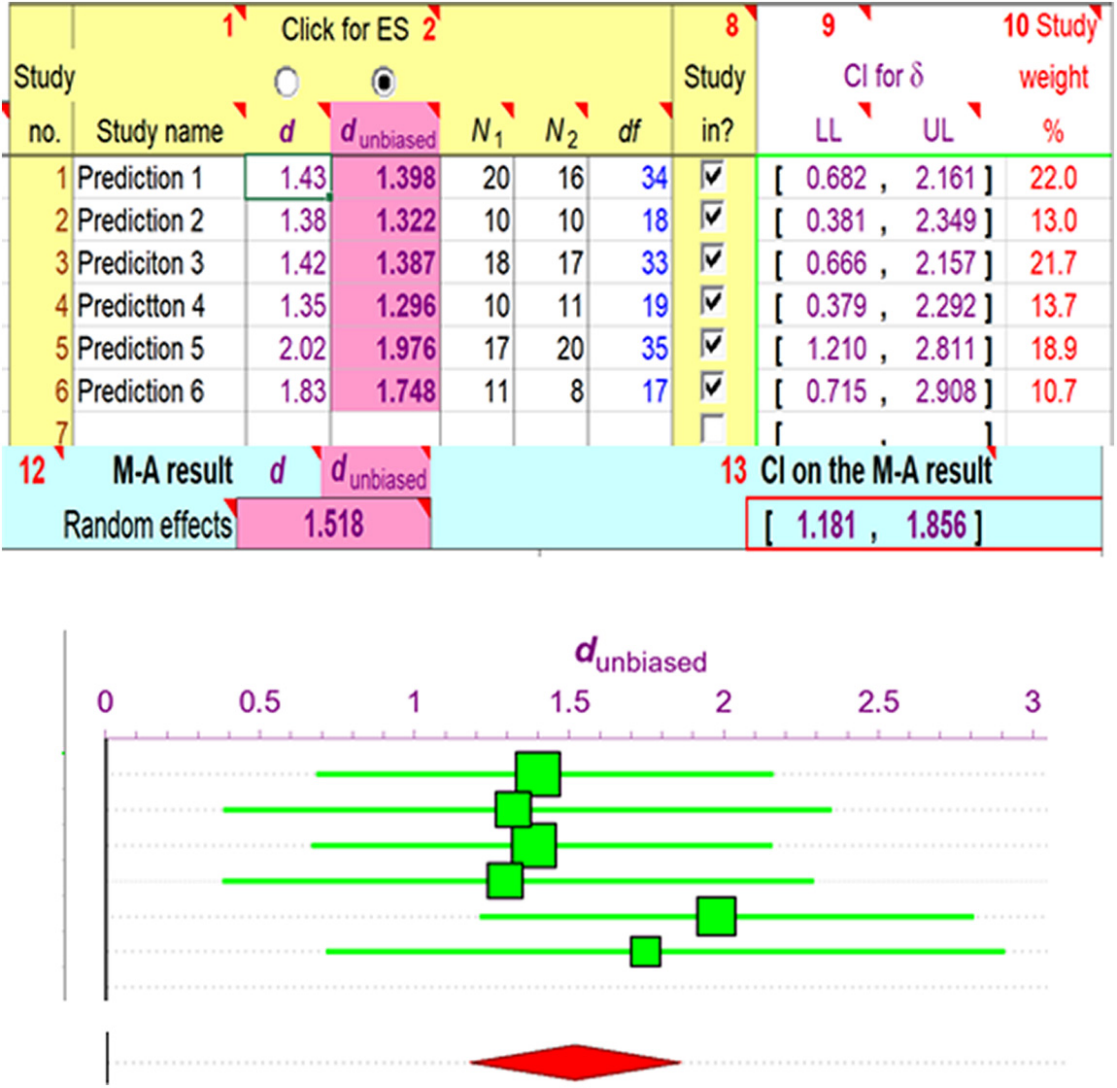
Meta-analysis of the effect sizes observed in the primary tests of the main hypothesis of Cen et al. (2018; *n* = 6; shown in Fig. 1). I obtained effect sizes by measuring the published figures and calculated Cohen’s *d* values with an on-line calculator: https://www.socscistatistics.com/effectsize/default3.aspx. Analysis and graphic display (screenshot) were done with ESCI (free at https://thenewstatistics.com/itns/esci/). Top panel shows individual effect sizes (corrected, d_unbiased_) for the tendency of small samples to overestimate true effect sizes (see Cummings and Calin-Jageman, 2017; pp 176–177), *N*s and degrees of freedom (df) of samples compared, together with confidence intervals (CIs) of effect sizes and relative weights (generated by ESCI and based mainly on sample size) that were assigned to each sample. Upper panel also shows mean effect size for random effects model and CI for mean. Bottom panel shows individual means (squares) and CIs for d_unbiased_ (square size is proportional to sample weight). The large diamond at the very bottom is centered (vertical peak of diamond) at the mean effect size, while horizontal diamond peaks indicate CI for the mean.

Fisher’s method is only one of a number of procedures for combining test results. To illustrate an alternative approach, I re-worked the assessment of Cen et al. (2018) as a meta-analysis (see Borenstein et al., 2007; Cummings and Calin-Jageman, 2017) of the effect sizes, defined by Cohen’s *d*, of the same predictions. Cohen’s *d* is a normalized, dimensionless measure of the mean difference between control and experimental values. I treated each prediction of the main hypothesis in Cen et al. (2018) as a two-sample independent comparisons test, determined Cohen’s *d* for each comparison, and conducted a random-effects meta-analysis (see Materials and Methods). Figure 3 shows effect sizes together with their 95% confidence intervals for each individual test, plus the calculated group mean effect size (1.518) and its confidence interval (1.181, 1.856). Effect sizes of 0.8 and 1.2 are considered “large” and “very large,” respectively, hence, an effect size of 1.518 having a 95% confidence interval well above zero is quite impressive and reinforces the conclusion reached by Fisher’s method, namely, that Cen et al.’s experimental tests strongly corroborate their main hypothesis.

The findings underscore the conclusions that (1) when evaluating the probable validity of scientific conclusions, it is necessary to take into account all of the available data that bear on the conclusion; and (2) obtaining a collection of independent experimental results that all test a given hypothesis constitutes much stronger evidence regarding the hypothesis than any single result. These conclusions are usually downplayed or overlooked in discussions of the reproducibility crisis and their omission distorts the picture.

To appreciate the problem, we can re-examine the argument that the PPV of much neuroscience is also low (Button et al., 2013). PPV is quantified as:

PPV = [(1−β) * R] / [((1−β) * R) + α)],

where R represents the “pre-study odds” that a hypothesis is correct, α is the *p* value, and 1-β is the power of the statistical test used to evaluate it. R is approximated as the anticipated number of true (T) hypotheses divided by the total number of alternative hypotheses in play, true plus false; i.e., R = T/(T + F). This argument depends heavily on the concept of “pre-study odds.” In the example of a “gene-screen” experiment (Ioannidis, 2005) that evaluates 1000 genes, i.e., 1000 distinct “hypotheses” where only one gene is expected to be the correct one (note that these are not true hypotheses, but it is simplest to retain the statisticians’ nomenclature here). R is ∼1/1000, and with a *p* value for each candidate gene of 0.05, PPV would be quite low, ∼0.01, even if the tests have good statistical power (≥0.8). That is, the result would have ∼1/100 chance of being replicated, apparently supporting the conclusion that most science is false.

Fortunately, these concerns do not translate directly to laboratory neuroscience work in which researchers are testing actual explanatory hypotheses. Instead of confronting hundreds of alternatives, in these cases, previous work has reduced the number to a few genuine hypotheses. The maximum number of realistic alternative explanations that I found in reviewing *The Journal of Neuroscience* articles was four and that was rare. Nonetheless, in such cases, R and PPV would be relatively high. For example, with four alternative hypotheses, R would be 1/4; i.e., ∼250 times greater than in the gene-screen case. Even with low statistical power of ∼0.2 and *p* value of 0.05, PPV would be ∼0.5, meaning that, by the PPV argument, replication of experimental science that tests four alternative hypotheses should be ∼50 times more likely than that of the open-ended gene-screen example.

Furthermore, PPV is also inversely related to the *p* value, α; the smaller the α, the larger the PPV. A realistic calculation of PPV should reflect the aggregate probability of getting the cluster of results. Naive joint probability considerations, Fisher’s method, or a meta-analysis of effect sizes, all argue strongly that the aggregate probability of obtaining a given group of *p* values will be much smaller than any one *p* value. Taking these much smaller aggregate probabilities into account gives much higher PPVs for multiple-part hypothesis-testing experiments. For example, Cen et al. (2018), as is very common, do not specify definite alternative hypotheses; they simply posit and test their main hypothesis, so the implied alternative hypothesis is that the main one is false; hence, R = 1/2. Applying *p* < 0.001, as suggested by both Fisher’s method and meta-analysis, to Cen et al.’s main hypothesis implies a PPV of 0.99, that is, according to the PPV argument, their primary conclusion regarding N-cadherin and PKD1 on synapse formation should have a 99% chance of being replicated.

Finally, we should note that these calculations incorporate the low statistical power reported by Button et al. (2013), i.e., 0.2, whereas actual power in many kinds of experiments may be higher. Cen et al. (2018) did not report a pre-study power analysis, yet *post hoc* power (as determined by G* Power software) for the six tests discussed earlier ranged from 0.69 to 0.91 (mean = 0.79), which, although much higher than the earlier estimate, is still underestimated. Power depends directly on effect size, which for the results reported by Cen et al. (2018) ranged from 1.38 to 2.02, and the version of G* Power that I used does not accept effect sizes >1.0. Thus, the higher levels of statistical power achievable in certain experiments will also make their predicted reliability dramatically higher than previously calculated.

## Discussion

To determine the validity and importance of a multifaceted, integrated study, it is necessary to examine the study as a whole. Neuroscience has no widely accepted method for putting together results of constituent experiments and arriving at a global, rational assessment of the whole. Since neuroscience relies heavily on scientific hypothesis testing, I propose that it would benefit from a quantitative way of assessing hypothesis-testing projects. Such an approach would have a number of benefits. (1) Typical papers are jammed full of experimental data, and yet the underlying logic of the paper, including its hypotheses and reasoning about them, is frequently left unstated. The use of combining methods would require authors to outline their reasoning explicitly, which would greatly improve the intelligibility of their papers, with concomitant savings of time and energy spent in deciphering them. (2) The reliability of projects whose conclusions are derived from several tests of a hypothesis cannot be meaningfully determined by checking the reliability of one test. The information provided by combining tests would distinguish results expected to be more robust from those likely to be less robust. (3) Criticisms raised by statisticians regarding the reproducibility of neuroscience often presuppose that major scientific conclusions are based on single tests. The use of combining tests will delineate the limits of this criticism.

Fisher’s method and similar meta-analytic devices are well-established procedures for combining the results of multiple studies of the “same” basic phenomenon or variable; however, what constitutes the “same” is not rigidly defined. “Meta-analysis is the quantitative integration of results from more than one study on the same or similar questions” (Cummings and Calin-Jageman, 2017; p. 222). For instance, it is accepted practice to include studies comprising entirely different populations of subjects and even experimental conditions in a meta-analysis. If the populations being tested are similar enough, then it is considered that there is a single null hypothesis and a fixed-effects meta-analysis is conducted; otherwise, there is no unitary null-hypothesis, and a random-effects meta-analysis is appropriate (Fig. 3; Borenstein et al., 2007; Cummings and Calin-Jageman, 2017). Combining techniques like those reviewed here have not, as far as I know, expressly been used to evaluate single hypotheses, perhaps because the need to do so has not previously been recognized.

Meta-analytic studies can reveal the differences among studies as well as quantify their similarities. Indeed, one off-shoot of meta-analysis is “moderator analysis” to track down sources of variability (“moderators”) among the groups included in an analysis (Cummings and Calin-Jageman, 2017, p. 230). Proposing and testing moderators is essentially the same as putting forward and testing hypotheses to account for differences. In this sense, among others, the estimation approaches and hypothesis-testing approaches clearly complement each other.

I suggest that Fishers’ method, meta-analyses of effect sizes, or related procedures that concatenate results within a multitest study would be a sensible way of assessing the significance of many investigations. In practice, investigators could report both the *p* values from constituent tests as well as an aggregated significance value. This would reveal the variability among the results and assist in the interpretation of the aggregate significance value for the study. Should the aggregate test parameters themselves have a defined significance level and, if so, what should that be? While ultimately the probability level for an aggregate test that a scientific community recognizes as “significant” will be a matter of convention, it might make sense to stipulate a relatively stringent level, say ≤0.001 or even greater, for any parameter, e.g., p_FM_, that is chosen to represent a collection of tests.

It is important that each prediction truly follow from the hypothesis being investigated and that the experimental results are genuinely independent of each other for the simple combining tests that I have discussed. (More advanced analyses can deal with correlations among *p* values; Poole et al., 2016.) There is a major ancillary benefit to this requirement. Ensuring that tests are independent will require that investigators plan their experimental designs carefully and be explicit about their reasoning in their papers. These changes should improve both the scientific studies and the clarity of the reports; it would be good policy in any case and the process can be as transparent as journal reviewers and editors want it to be.

Besides encouraging investigators to organize and present their work in more user-friendly terms, the widespread adoption of combining methods could have additional benefits. For instance, it would steer attention away from the “significance” of *p* values. Neither Fisher’s method nor meta-analyses require a threshold *p* value for inclusion of individual test outcomes. The results of every test of a hypothesis should be taken into account no matter what its *p* value. This could significantly diminish the unhealthy overemphasis on specific *p* values that has given rise to publication bias and the “file drawer problem” in which statistically insignificant results are not published.

Use of combining tests would also help filter out single, significant-but-irreproducible results that can otherwise stymie research progress. The “winner’s curse” (Button et al., 2013), for example, happens when an unusual, highly significant published result cannot be duplicated by follow-up studies because, although highly significant, the result was basically a statistical aberration. Emphasizing the integrated nature of most scientific hypothesis-testing studies will decrease the impact of an exceptional result when it occurs as part of a group.

Of course, no changes in statistical procedures or recommendations for the conduct of research can guarantee that science will be problem free. Methods for combining test results are not a panacea and, in particular, will not curb malpractice or cheating. Nevertheless, by fostering thoughtful experimental design, hypothesis-based research, explicit reasoning, and reporting of experimental results, they can contribute to enhancing the reliability of neuroscience research.

Recently, a large group of eminent statisticians (Benjamin et al., 2018) has recommended that science “redefine” its “α” (i.e., significance level), to *p* < 0.005 from *p* < 0.05. These authors suggest that a steep decrease in *p* value would reduce the number of “false positives” that can contribute to irreproducible results obtained with more relaxed significance levels. However, another equally large and eminent group of statisticians (Lakens et al., 2018) disagrees with this recommendation, enumerating drawbacks to a much tighter significance level, including an increase in the “false negative rate,” i.e., missing out on genuine discoveries. This second group argues that, instead of redefining the α level, scientists should “justify” whatever α level they choose and do away with the term “statistical significance” altogether.

I suggest that there is third way: science might reserve the more stringent significance level for a combined probability parameter, such as p_FM_. This would provide many of the advantages of a low *p* value for summarizing overall strength of conclusions without the disadvantages of an extremely low *p* value for individual tests. A demanding significance level for conclusions derived from multiple tests of a single hypothesis would help screen out the “false positives” resulting from single, atypical test results. At the same time, a marginal or even presently “insignificant” result, would not be discounted if it were an integral component of a focused group of tests of a hypothesis, which would help guard against both the problem of “false negatives” and an obsession with *p* values.

**Table 1:** Analysis of *The Journal of Neuroscience* Research Articles

Start	End	Hyp-E	Hyp-I	Alt Hyp	# Tests	Support	Reject	Disc	Ques	Comp
32	50	X			6	X				
51	59		X	2	5	X	X			
60	72		X		7	X	X			
74	92		X		7	X				
93	107		X		6	X				
108	119								X	
120	136		X		7	X				
137	148		X	3	8	X	X			
149	157	X		2	5	X	X			
158	172		X	2	8	X	X			
173	182	X		2	5		X			
183	199	X			9	X				
200	219							X		
220	231								X	
232	244	X			7	X				
245	256	X		1	7	X	X			
263	277	X		1	7	X	X			
278	290								X	
291	307		X		7	X				
308	322		X	1	7	X	X			
322	334		X		7	X				
335	346								X	
347	362							X		
363	378		X		6	X				
379	397	X		1	8	X	X			
398	408									X
409	422		X	1	4	X				
423	440	X		3	8	X	X			
441	451	X		2	9	X	X			
452	464		X		6	X				
465	473	X			8	X				
474	483	X			9	X				
484	497	X		1	9	X	X			
498	502								X	
518	529		X		8	X				
530	543									X
548	554		X	1	8	X				
555	574		X		9	X				
575	585	X			8		X			
586	594							X		
595	612	X		1	3	X	X			
613	630		X	1	7	X	X			
631	647		X		8	X				
648	658	X			6	X				
659	678	X		3	5	X				
679	690	X.			5		X			
691	709									X
710	722									X
723	732		X	1	5	X	X			
733	744									X
										
745	754	X		1	4		X			
755	768		X		5	X				

Classification of research reports published in *The Journal of Neuroscience*, vol. 38, issues 1–3, 2018, identified by page range (*n* = 52). An x denotes that the paper was classified in this category. Categories were: Hyp-E: at least one hypothesis was fairly explicitly stated; Hyp-I: at least one hypothesis could be inferred from the logical organization of the paper and its conclusions, but was not explicitly stated; Alt-Hyp: at least one alternative hypothesis in addition to the main one was tested; # Tests: is an estimate of the number of experiments that critically tested the major (not subsidiary or other) hypothesis; Support: the tests were consistent with the main hypothesis; Reject: at least some tests explicitly falsified at least one hypothesis; Disc: a largely “discovery science” report, not obviously hypothesis-based; Ques: experiments attempted to answer a series of questions, not unambiguously hypothesis-based; Comp: mainly a computational modeling study, experimental data were largely material for model.

## Synthesis

Reviewing Editor: Leonard Maler, University of Ottawa Decisions are customarily a result of the Reviewing Editor and the peer reviewers coming together and discussing their recommendations until a consensus is reached. When revisions are invited, a fact-based synthesis statement explaining their decision and outlining what is needed to prepare a revision will be listed below. The following reviewer(s) agreed to reveal their identity: Raymond Dingledine.

Dear Dr Alger

Your revised manuscript is much improved. One reviewer has requested some minor revisions (below). I have also read the revised ms in detail and fully agree with this reviewer. I think this Ms can potentially be very relevant to the design of statistical analyses of neuroscience papers.

I have not been able to get a second reviewer and I think, in light of the comments of the one reviewer and my own careful review of the Ms, that it will not be necessary to do so.

Leonard Maler

Reviewer comments:

My initial comments have all been addressed. However, the extensive new text raises a few additional issues.

line 129: “random” - an unfortunate use of the word, unless Brad actually used a randomizing technique to select the paper.

line 184-85 “If the tests were done at p<.05....” I get the logic of this, with the analogy of the odds of flipping 5 heads in a row for example, but it doesn’t seem entirely right. For example if the investigator were to select p<.8 for each 2-sided test of an hypothesis, and perform 20 such tests, the random chance that all 20 tests would be consistent with the hypothesis is 0.012. Fisher’s method takes care of this problem, with chi2=8.9 and 40 DF the combined pFM-value is >99.9%, ie not in support of the hypothesis. It would be worth pointing this issue out to the reader unless my logic is faulty.

line 249: “i” should be subscript for clarity

line 358: “mutli"

## Author Response

Dear Dr. Maler,

I was encouraged to learn that both reviewers found merit my manuscript that might, if suitably revised, allow it to be published. I thank both for their thoughtful comments and suggestions and believe that responding to them has enabled me to improve the paper in many ways.

The MS was submitted, at the recommendation of Christophe Bernard, as an Opinion piece and I felt constrained by the journal’s “2-4 page,” limit on Opinions. This revision includes a fuller description of my arguments, a worked example, as well as additional discussion that the original MS lacked. I suggested Fisher’s Method as an example of a combining approach that offers a number of benefits to interpreting and communicating research results. The Method has been well-studied, is intuitively easy to grasp and, provided that the combined p-values are independent, is robust. Most importantly, I believe that its use would have the salutary effect of obliging authors to be more explicit about their reasoning and how they draw their conclusions than they often are. However, there is nothing unique about Fisher’s Method, and I now include a meta-analysis of effect sizes of the identical results that I used with Fisher’s Method, as well as citations to alternative methods, to make this point. I have re-titled the paper to reflect the de-emphasis of the method, as I believe is consonant with the critiques of the MS.

Reviewer 1 raised several profound objections that, in the limit, would call into question the validity of most published neuroscience research. Because these are unquestionably complex and significant issues, I’ve addressed them in detail here but, because they go far beyond the confines of my modest Opinion, have only touched on them briefly in the text. The text has been thoroughly revised with major changes appearing in bold.

General remarks

I am afraid that my presentation of Fisher’s Method overshadowed my overarching goal, which was to call attention to weaknesses in how scientists present their work and how published papers are evaluated, I used the reproducibility crisis to call attention to both problems. I believe that, while testing scientific hypotheses is the bulwark of much research, as a community we do not think about or communicate as clearly as we could, and that the reliability of scientific hypothesis-testing work is, as a result, under-estimated by statistical arguments.

The idea of using Fisher’s Method, essentially a meta-analytic approach, is a novel extension of current practice, and I’ve clarified and moderated my proposal in this regard. The changes do not alter the fundamental argument that having multiple tests focused on a single hypothesis can lead to stronger scientific conclusions than less tightly organized collections of results.

When investigators do multiple different tests of a hypothesis, they expect the reader to put the results together intellectually and arrive at a coherent interpretation of the data. Yet there is no standard objective method for quantitatively combining a group of related results. Both Reviewers allude to the problem of the complexity of published papers. Reviewer 1 calls the Cen et al. (2018) paper a “tangle” of results (I counted 116 comparisons in the figures) and doubts that any coherent interpretation of them is to be had. Likewise Reviewer 2 notes that Bai et al. (2018) lists 138 p-values, and asks how they could be interpreted.

The Reviewers’ vivid descriptions of the confusing ways in which papers are presented confirms the need for improvements in current practices. I believe that improvements are possible and that focusing attention on the hypotheses that are usually present in many publications can be a starting point for change.

The Reviewers’ comments underscore the deficiencies in the current system, which is sadly lacking in impetus for rigor of communication. Placing the onus of discerning the underlying logic that shapes a project exclusively on readers is unreasonable. I believe papers can be made much more user-friendly. Authors should state whether they have a hypothesis and which of results test it. They should tell us plainly their conclusions are justified and why. I suspect that merely telling people to change their ways would have little effect. An indirect approach that offers investigators the ability to apply a solid, objective way of summarizing their results might be a step in right direction.

Reviewer 1

The Reviewer raises several issues, but two are most important: a request for a worked example, which I now provide, and the “file drawer problem,” in which results that do not meet a prescribed significance level, usually p<0.05, are not reported, but left in a file drawer, which skews the literature towards positive results. My proposal can contribute to alleviating this problem.

1. A worked example; Cen et al (2018).

Cen et al. state (p. 184):

”... we proposed that N-cadherin might contribute to the “cell- cell adhesion” between neurons under regulation of PKD.”

Further, “In this work, we used morphological and electrophysiological studies of cultured hippocampal neurons to demonstrate that PKD1 promotes functional synapse formation by acting upstream of N-cadherin.”

In the last paragraph of the Discussion (p. 198) they conclude:

"Overall, our study demonstrates one of the multiregulatory mechanisms of PKD1 in the late phase of neuronal development: the precise regulation of membrane N-cadherin by PKD1 is critical for synapse formation and synaptic plasticity, as shown in our working hypothesis (Fig. 10).” The bolded quotes express, respectively, their main hypothesis, the methods they use to test it, and an overview of their conclusions about it. After testing the main hypothesis, they tested related hypotheses that each had its own specific predictions. All of the findings are represented in their model.

In brief (details in Methods section of MS) to analyze the paper I counted as actual predictions only experiments which were capable of falsifying the hypothesis. Non-quantifiable descriptive experiments contributed logically to the hypothesis test, but obviously did not count quantitatively. They authors also included many control experiments and other results which served merely to validate a particular test, and I do not include these either. To simplify the example and avoid possibly including non-independent tests, if more than one manipulation tested a given prediction, say against a common control group, I counted only the first one. Note that this is an extremely conservative approach, as including more supportive experiments in meta-analyses typically strengthen the case.

For example, Cen et al. (2018) tested the following predictions of their main hypothesis:

a. Prediction: PKD1 increases synapse formation. Test: Over-expression (OE) of hPKD1 increases spine density (p<0.001); dominant negative (DN)-hPKD1 decreases spine density (p<0.001).

b. Prediction: PKD1 increases synaptic transmission. Test: OE of hPKD1 increases mEPSC frequency (p<0.05); DN-hPKD1 decreases mEPSCfrequency (p<0.01).

c. Prediction: PKD1 regulates N-cad surface expression. Test: Surface biotinylation assay. OE hPKD1 increases N-cad (p<0.05); DN-hPKD1 decreases N-cad (p<0.001).

d. Prediction: PKD1 acts upstream of N-cad on synapse formation. Test: knock-down of PKD1 is rescued by OE of N-cad; spine density (p<0.001) and mEPSC frequency (p<0.01).

I believe a strong case can be made that each prediction does follow logically from the main hypothesis. Furthermore, although the subsidiary subsequent hypotheses are linked to the main one, they are clearly separate hypotheses and not predictions of it; no matter what the outcomes of testing them are, they do not directly reflect on the truth or falsity of the main hypothesis.

Thus, the authors go on to identify specific sites on N-cad that PKD1 binds and phorphorylates and to test the hypothesis that these sites are critical for the actions of PKD1 on N-cad. They also investigate β-catenin as a binding partner for N-cad and test the hypothesis that this binding is promoted by PKD1. At the end, they test the hypothesis that N-cad influences LTP and acts pre-synaptically. These are distinct, though related, hypotheses that feed into the, authors’ summary hypothesis. I believe the structure of Cen et al.’s paper, their procedures, and conclusions follow a reasoned, logical plan, which is not always as explicitly delineated as it could be, but is typical of most JNS papers (see also analysis of Bai et al., below). I now include diagrams of the complete structure of the paper, together with a detailed diagram of the logic of the main hypothesis in Figures 2 and 3.

2. My use of Fisher’s Method.

In his 1932 text R.A. Fisher states:

"When a number of quite independent tests of significance have been made, it sometimes happens that although few or none can be claimed individually as significant, yet the aggregate gives an impression that the probabilities are on the whole lower than would often have been obtained by chance. It is sometimes desired, taking account only of these probabilities, and not of the detailed composition of the data from which they are derived, which may be of very different kinds, to obtain a single test of the significance of the aggregate, based on the product of the probabilities individually observed.” (Italics added.)

The Reviewer asks if all tests in Cen et al. relate to “the same hypothesis?” This question has two answers: one trivial, one substantial. The Reviewer also asks about “all available evidence.”

a. The “same hypothesis?” The answer is “no” in the trivial sense: there is more than one hypothesis in the paper. Yet the paper is organized into clusters of experiments that each do test “the same hypothesis.” As shown above, many experiments test predictions of the main hypothesis.

In a more interesting sense, the answer is “yes,” each group of experiments does a single hypothesis. First, it is critical to distinguish between, “statistical” and “scientific” hypotheses, as they are not the same thing (see Alger, 2019). The scientific hypothesis is a putative explanation for a phenomenon; the statistical hypothesis is part of a mathematical procedure, e.g., for comparing results. Certainly, when combining tests from different kinds of experiments, e.g., electrophysiological recordings and dendritic spine measurements, then the identical statistical null hypothesis cannot be at issue, as the underlying populations are entirely different. But combined analyses do not require the identical null hypotheses (see, e.g., overview in Winkler et al. 2016). Or consider that a standard random-effects meta-analysis is specifically designed to include different populations of individuals and different experimental conditions. In fact, commonly, “... a meta-analysis includes studies that were not designed to be direct replications, and that differ in all sorts of ways.” (Cummings and Calin-Jageman, p. 233). Hence, the “same hypothesis” cannot mean the same statistical null hypothesis. Actually. Fisher’s Method tests a “global null” hypothesis not a single null and that is why it can accommodate the results of many different experimental techniques. Indeed, this approach allows combining in a rational way groups of results in a way that agrees with common usage and reasoning.

b. Are all tests a “fair and unbiased sample of the available evidence?” In a combining method, authors identify which of their experiments test the hypothesis in question and include all of those test results in their calculations. There is no selection process; simply include all of the relevant tests that were done.

3. The “file drawer problem"

The Reviewer alludes several times to the “file-drawer” problem. As I emphasize in original and revised MSs, Fisher’s Method does not presuppose or require a particular threshold p-value for significance, if the p-value for a test is 0.072, then that is what goes into calculating pFM. Therefore, both Fisher’s Method and analogous meta-analytic approaches explicitly and directly work against the file-drawer problem by calling for the reporting of all relevant data. Combining methods can help ameliorate, not exacerbate, the file drawer problem.

In the specific context of Cen et al. (2018) is there internal evidence for the problem?

a. According to Simonsohn et al. (2013) the “p-curve,” i.e., the distribution of p-values, for a set of findings is highly informative. The basic argument is that, if investigators are selectively sequestering insignificant results, e.g., those with p-values > 0.05, then there will be an excess of p-values which just reach significance, between 0.04 and 0.05 because, having achieved a significant result, investigators lose motivation to try for a more significant one. There will be a corresponding dearth of results that are highly significant, e.g. {less than or equal to} 0.01. In contrast, legitimate results will tend to have an excess of highly significant results have the opposite pattern, that is, a large excess of highly significant results and far fewer marginal ones. I did a census of the p-values (n = 114) in Cen et al.’s figures and found there were 52 at {less than or equal to} 0.001, 20 between 0.01, 18 at 0.05 and 24 non-significant at 0.05. Thus the p-value pattern in this paper is exactly opposite to the expectation of the file-drawer problem.

b. Too small effect sizes and-or low power would be inconsistent with the pattern of highly significant results and might suggest “questionable research practices.” To see if the effect sizes and post-hoc power calculations (a priori values were not given) in Cen et al.’s paper were consistent with their p-values, I measured means and standard errors roughly from the computer displays of their figures. I determined effect sizes with an on-line effect-size calculator (https://www.socscistatistics.com/effectsize/default3.aspx). The mean effect sizes (Cohen’s ds) was 1.518 (confidence interval from 1.181 to 1.856). I used G*power (http://www.psychologie.hhu.de/arbeitsgruppen/allgemeine-psychologie-und-arbeitspsychologie/gpower.html) and found that the post-hoc power of these tests ranged from 0.69 to 0.92 (mean = 0.79). Although these power values are very good, they are underestimates, because G*power does not accept an effect size greater than 0.999, i.e., less than the real values. Since power increases with effect size, the actual power of Cen et al.’s tests were even higher than estimated. Finally, I carried out a meta-analysis of measured effect sizes with the free software program ESCI (available at https://thenewstatistics.com/itns/esci/; Cummings and Calin-Jageman, 2017). As Figure 4 in the MS shows, the mean effect size (vertical peak of the diamond at the bottom) falls within the cluster of effect sizes and has a 95% confidence interval that is well above zero. These large values are consistent with the results that Cen et al. claim. In short, there is no obvious reason to think that proceeding to analyzing with a combined test would no be legitimate.

c. The Reviewer implies that the prevalence of positive results in, e.g., JNS papers, is prima facie evidence either of “incredible predictive ability” or bad behavior on the part of investigators. This seems to be an unduly harsh or cynical view of our colleagues and a very depressing slant on our field, I would like to suggest other plausible explanations:

c.i. Ignorance. According to a survey that I conducted (Alger, 2019; Fig. 9.2A, p. 221), the majority of scientists (70% of 444 responses) have had {less than or equal to} 1 hour of formal instruction in the scientific method and scientific thinking. This mirrors my own educational background, and I strongly suspect that many neuroscientists are simply unaware of the importance of reporting negative or weak results.

c.ii. Imitation. Authors, particularly younger ones, mimic what they read about and see in print. It is a vicious cycle that should be broken, but in the meantime we shouldn’t be surprised if individuals who see others reporting only positive results do the same.

c.iii. Distorted scientific review and reward system. The simple fact is that referees, journal editors, and granting agencies hold the power to say what gets published and, therefore, rewarded by grants, promotions, speaking engagements, etc. If the literature is skewed, the primary responsibility lies with these groups. Authors have no choice but to follow their lead.

c.iv. The conundrum of pilot data. This crucial issue often gets little attention in this context,, so I’ll expand on it. Briefly, I believe that the most common data found in file drawers are not well-controlled, highly powered, definitively negative studies but, rather, small, weak, inconclusive clutches of pilot data that just seemed “not worth following up.”

These are the kinds of data that reviewers would probably reject as meaningless, and herein lies the conundrum. If an investigator wants to abandon an apparently unpromising line of investigation and also wants to avoid committing the file-drawer offense, what to do? Continue work to achieve a decisive result with a small, variable effect size? This could require large numbers of tests, many subjects etc., to achieve adequate power for a result of possibly dubious value, or even a situation in which the required ‘n’ becomes so “impractically and disappointingly large” (e.g., Cummings and Calin-Jageman, 2017; p. 285) it is infeasible to proceed.

Like most of my colleagues, I have faced this issue and made what I thought was a rational and defensible decision to stop throwing scarce and hard-won taxpayer dollars down the drain and wasting my and most co-workers valuable time and effort on a dead-end project. But, and this is absolutely crucial for the argument, strictly speaking my decision was unjustified. Because I found, say, the effect sizes of drug X, were small and my n’s were low, the power of my pilot tests did not permit me to reject the hypothesis that drug X was effective, with confidence. It appears that an extreme “report all data” standard could mean that we can’t do a study without being committed to turning it into a full investigation. Probably the file drawer problem would solved or drastically reduced by abolishing all pilot studies. But everyone agrees that this is nonsense; pilot studies that are “hardly ever for reporting” are a vital, integral part of science (Cummings and Calin-Jageman, p. 261).

An alternative to banning pilot studies might be to place inconclusive data into an unreviewed repository, such as bioRxiv. Of course, this would transform much of the “file-drawer problem” into a “bioRxiv problem’” and not get more negative partial results published in refereed journals. The literature would still be dominated by positive results. A major advance would be to nurture much needed respect for thoroughgoing, rigorously obtained negative data, a goal that I heartily support, because good hard negative data play the lead role in rejecting a false hypothesis. This worthy goal is independent of the pilot data conundrum, the question of using Fisher’s Method, or other forms of meta-analyses along the lines I suggest.

d. The Reviewer concludes, “Finally, and most importantly, it is highly unlikely that Cen et al. reports all of the evidence collected for this project [...] without testing at least a couple of other possibilities.” But this leads back to the concern about pilot studies; indeed Cen et al., might well have done unreported pilot studies.

In sum, whether it is reasonable or not to use Fisher’s Method, or to include expanded sets of would-have-been pilot data, are critical though nrelated questions. Mixing them together will not help us answer either one.

4. Do I either misunderstand Fisher’s Method, “most neuroscience research,” or both?

I have explained my rationale for suggesting Fisher’s Method and have now included another method of meta-analysis which leads to the same conclusions as that Mether. I have carefully explained, documented, and analyzed the neuroscience research that my proposal is intended for. By design, my proposal only applies to multiple-prediction hypothesis-testing studies, as others are ineligible for combining methods.

5. How did I code the papers and was my system reliable?

The Reviewer asks about the “coding” that I used to classify the papers as hypothesis-testing or not. I did not code the papers in a rote, mechanical way. Instead, I read the papers carefully and judged them as sensibly as I could in concert with other factors:

I was mindful of the results of a survey that I conducted of hundreds of scientists (Alger, 2019, Fig. 9,7, p. 226), in which a majority (76%) of respondents (n = 295) replied that they “usually” or “always” stated the hypothesis of their papers. So I assumed that the papers had a logical coherence and looked for it.

To address the issue of reliability, I re-analyzed the 52 JNS papers from 2018 Inasmuch as the nearly all of them are well outside my area of expertise, and it had been over 9 months since I went over them, I had very little retention of their contents (or even ever having seen them before!). I re-downloaded all the papers to start with fresh pdfs and analyzed them again without looking at my old notes. The original- and re-analyses agreed reasonably well.

In the most important break-down, I initially classified 42/52 (80.8%) as hypothesis testing, on re-analysis, I classified 38/52 (73.1%) this way. Of the 42 initially classed as hypothesis-testing in the re-analysis, I categorized 35 (83.3%) this way. The distribution of explicit to implicit hypotheses was 20 and 22, originally, and 19 and 19 in the reanalysis. The average number of experimental tests per hypothesis-testing paper was 7.5 {plus minus} 2.26 (n = 42, CI, 6.187,8.131) initially, and 6.08 {plus minus}2.12 (n = 38, CI, 5.406, 6.754) on re-analysis (Cohen’s d = 0.648). It appears that my judgments were fairly consistent, with the exception of my tallies of the average number of critical experiments/paper. In this case, prompted by the Reviewers’ comments, I omitted control and redundant experiments that I had initially included and focused on key manipulations that were most directly aimed at testing the main hypothesis. This change does not alter the impact of the analysis. In preparing the revision, I have re-reviewed key discrepancies between original and re-analysis and reconciled them in the two analyses and updated Table 1 to reflect my best judgments.

Naturally, there may be honest disagreements in judgments about whether an experiment does test a legitimate prediction of a hypothesis, whether it actually follows necessarily and, hence, could really falsify it, etc.. This is why I do not classify Cen et al.’s prediction that PKD1 enhances LTP as following from their main hypothesis. The known linkages between mechanisms of synapse formation and LTP do not allow one to deduce that PKD1 must enhance LTP. That is, if PKD1 did not enhance LTP, their main hypothesis would not be threatened. Of course, the analysis underlying that classification rating in the Table is necessarily less exhaustive than my treatments of the Cen et al., (2018) and Bai et al. (2018) papers.

Regardless of the precise details, I think that it’s clear that the majority of research published in JNS relies on some form of hypothesis testing and draws its conclusions from multiple lines of evidence. Despite the good agreement in my two analyses, I stress that the main points that I wanted to make were, firstly, that the hypothesis-testing science that dominates the pages of JNS, is entirely distinct from the studies that base their major conclusions on single experiments that the statisticians focus on. And, secondly, that scientific authors should be inspired to make the presentation of their work more readily interpretable to readers.

The fact that many papers are beset by needless complexity is a shortcoming that I suspect both Reviewers would agree exists. Ideally, by calling attention to how scientific papers are organized, my approach will help encourage authors to be more forthright in explaining and describing their work, its purpose and logic.

I think that what is frequently lacking in scientific publications is a clear outline of their underlying logical structure. Our current system tolerates loose reasoning and vagueness and authors sometimes throw all kinds of data into their paper and leave it to the rest of us to figure it all out. I find this sort of dis-organization in a paper deplorable and suspect the Reviewers would agree. Where we may disagree is in our interpretations of the mass of data that is often presented. I believe that poor communication practices do not imply poor science. Rather they reflect the factors-deficient training, imitation, improper rewards-that I’ve alluded to, rather than absence of structure. Reviewer 1 says, “there is no easy way,” of untangling results, and I agree. Yet, deficiencies in presentation skills are correctible with training and encouragement from responsible bodies, mentors, and supervisors. Requiring authors to state their reasoning explicitly is exactly the kind of benefit that procedure such as Fisher’s Method could help foster.

6. Does Boekel et al. (2015) seriously undermins confidence in neuroscience generally?

The main point that Boekel et al. make is that, when using pre-registration standards-publishing their intended measurements, analysis plan, etc., in advance, they got very different results than had been reported in a series of original studies they tried to directly replicate. However, this study, though evidently carefully carried out, need not destroy faith in neuroscience research for at least two reasons: a) the research investigated by Boekel et al. is not representative of a great deal of neuroscience research such as in JNS. b) As a form of meta-science, Boekel et al.’s results themselves are subject to criticism, some of which I offer below.

a. Boekel et al., tried to replicate five cognitive neuroscience studies, comprising 17 sub-experiments, which were mainly correlational comparisons of behavioral phenomena, e.g. response times or sizes of social networks, with MRI measurements of gross brain structures. Their replication rate was low: only 1/17 (6%) studies met their criterion for replication. Although one way of assessing replicability is to ask whether or not the mean of the replicating study falls within the confidence interval of the original study (e.g. Calin-Jageman and Cummings, 2019). The data of Boekel et al. show (their Fig. 8) that this obviously happened in 5 cases, and was borderline in 2 others. In this sense, the replicators got “the same results” in 5/17 (29%) or 7/17 (41%) experiments, which are lower than the 83% expected (Calin-Jageman and Cummings, 2019), though clearly greater than 6%. In any event, the message of Boekel et al. was heavily dependent on the outcome of a single test; e.g. the predicted relationship between the behavior measure and an MRI measurement.

In contrast with the type of experiment, both Reviewers of my MS found that a randomly selected JNS paper reported a highly complex set of experiments that supported its conclusion. Such papers cannot be meaningfully assessed by testing one piece of evidence. No single experiment per se in either Cen et al. or Bai et al., is so crucial that the main message of the papers would be destroyed if it were not replicated. Failure of an experiment could mean that the hypothesis is false, or it could point to a variety of alternative explanations for the failure.

c. Boekel et al.’s study is quite small, including only five replication attempts and two are from the same group (Kanai and colleagues), which raises broad concerns about the degree to which it represents neuroscience at large, as well as issues of sample independence in this study. More flags are raised by the fact that, in all, Boekel et al. examined 17 correlational experiments from the five studies. The MRI tests were done on limited groups of subjects, i.e., on average, > 3 datasets were obtained from each subject. Can these be considered 17 independent studies and, if not, to what extent this would affect the validity of the conclusions?

Boekel et al.’s work was done in their home country, the Netherlands, and yet three of the five studies involved measures (e.g., social media behavior) which could well show a cultural influence. In their Discussion, the authors consider these and other factors that could have influenced their study and indicate that further tests are required to dig deeper..

d. Reviewer 1’s comments about not using “all available data” seem especially germane to Boekel et al., who acknowledge that the experiments they chose to replicate for their meta-analysis were not selected randomly or exhaustively, but were specifically chosen, “...from the recent literature based on the brevity of their behavioral data acquisition.” However justifiable this rationale might be, their selection process runs counter to the importance of using “all available evidence” in a broad meta-analytic context.

My concern about the generalizability of Boekel et al.’s results is further heightened by a number of additional peculiarities of their paper:

Remarkably, the first paper that Boekel et al., attempt and fail to replicate (Forstman et al., 2010) is from their own laboratory (Forstmann is the senior author on Boekel et al.). Moreover, Forstmann had already replicated their own result in 2010, before the 2015 study. In 2010, the authors report replications of measurements of Supplementary Motor Area (SMA) involvement in their behavioral test, which involves a psychological construct, the LBA (“Caution”) parameter, which purports to account for a “speed-accuracy” trade off that their subjects perform when responding quickly to a perceptual task. There are a number of worrisome features of these studies: In the original study (Forstmann 1) they show a highly significant and precise (p=0.00022) correlation, r = 0.93, between the SMA measure and the Caution parameter. Likewise, in the 2010 replication, they find r = 0.76 (p=0.0050). In 2015, Boekel et al. found r=0.03, and no p-value was reported (the Bayes factor provided some support for the null hypothesis of zero correlation). It is unlikely that simple sampling error can account for the marked differences among the three tests and, as noted, the authors discuss several possibilities that could account for systematic error..

It is worth mentioning in this context that Boekel et al. (2015) has been challenged on technical grounds (Boekel et al. responded in 2016; Cortex 74:248-252). Nevertheless, in 2017 (M.C. Keuken, et al. Cortex, 93:229-233), also from Forstmann’s laboratory, published a lengthy “Corrigendum” in which they corrected a “mistake in the post-processing pipeline” and a number of other errors in Boekel et al. (2015) which did not, however, alter their original conclusions.

(As a side note, according to Google Scholar, Forstmann et al. (2010) has been cited a respectable 328 times and Boekel et al. (2015) 154 times (as of 4/4/20). Apparently, Forstmann et al. (2010) was wrong yet this paper does not appear to have been retracted.)

For all of these reasons, I do not think that the findings of Boekel et al. (2015) can be fairly generalized to question the validity of neuroscience research as a whole.

As regards more extensive replicability studies, I have extensively reviewed the Reproducibility Study: Psychology (RPP) by the Open Science Consortium, led by Nosek that attempted to reproduce 100 psychology studies (Alger, 2019, Chp. 7). In brief, the reproducibility rate of the RPP varied from 36 - 47%, low, but again much higher than Boekel et al. (2015) found. In the book, I also cover critical reviews of the RPP, which identified a range of technical issues, including faithfulness of replication conditions, statistical power calculations etc., leading to the conclusion that, while reproducibility was probably lower than would be ideal, the observed rates were not so far from the expected rates as warrant declaring a “crisis.”

Conclusion of response to Reviewer 1.

I regret having conveyed an unrealistically “sunny” picture of neuroscience (which I do not hold) and have tried to correct this impression in the text. I do believe that the reliability of neuroscientific research is as poor as extreme views have it, that reliability is enhanced when rigorous, well-controlled, statistically sound, procedures are applied to testing hypothesis with a battery of logically-entailed predictions. I drew on the statisticians’ construct of PPV, to show that my view is consonant with the statistical arguments. The process of presenting experimental results logically, identifying scientific hypotheses, and experiments that test them can yield considerable benefits to neuroscience. The concept of combining results objectively using Fisher’s Method, meta-analysis, or perhaps other techniques, can bring needed attention to this often overlooked topic.

Reviewer 2

The Reviewer points to the paper by Bai et al. (2018, JNS, pp. 32-50) to raise questions of the independence of tests, conceptual focus of papers, and “pre-study odds.” In response, I have discussed the caveats of Fisher’s Method, reduced its prominence and, as the Reviewer recommends, added an alternative method of meta-analysis to decrease emphasis on Fisher’s Method and the PPV argument.

Reviewer 2 echoes Reviewer 1’s concerns about applying a method like Fisher’s given the complexity of the literature. To respond to this concern, I have analyzed Bai et al., as I did Cen et al.. although I do not include this analysis in the MS. As the Reviewer infers, there is more than one hypothesis in the paper; a central one as well as ancillary hypotheses that are linked to it, but are not predicted by the main one. Despite its complexity however, I believe there is a relatively straightforward logical structure to the paper.

1. Hypothesis tests in Bai et al. (2018)

The main hypothesis of this paper is that circular RNA DGLAPA (circDGLAPA) regulates ischemic stroke outcomes by reducing the micro RNA, miR-143, levels and preserving blood-brain barrier (BBB) integrity. The authors test several predictions of this hypothesis:

a. Prediction: miR-143 levels should be elevated in ALS and stroke models. Test: measure miR-143 level in ALS patients and tMCAO mice. (Fig. 1)

b. Prediction: miR-143 levels should be related to degree of stroke damage: Test: compare infarct size in tMCAO WT and miR-143 knock-down (miR-143+/-) mice. (Fig. 2)

c. Prediction: tMCAO-induced loss of cerebrovascular integrity will be reduced in miR-143+/- mice. Test: BBB permeability in WT and miR-143+/- tMCAO models. Levels of tight junctional proteins (TJPs) are decreased by miR-143. (Fig. 2)

d. Prediction: circDGLAPA should bind to miR-143 and be decreased in stroke models. Test: measure circDGLAPA levels in ALS patients and tMCAO mice. Biochemical assays and cellular co-localization of circDGLAPA and miR-143. (Figure 3)

e. Prediction: overexpression of circDGLAPA should be neuroprotective in tMCAO mice. Test: infarct size, BBB permeability, and TJP protein measurements in control and overexpression of circDGLAPA conditions. (Figs. 4, 5)

I believe these are all direct predictions of the main hypothesis; falsification of any of them would call for its rejection or revision. After testing the main hypothesis, Bai et al. go on to test two related mechanistic hypotheses about how miR-143 damages the BBB. These hypotheses are that: miR-143 regulates the transition of endothelial cells into mesenchymal cells (EndoMT), and that that it does so by targeting HECTD1.

Inasmuch as the outcomes of the two mechanistic explanations cannot affect the truth or falsity of the main hypothesis, I do not classify them as integral to it but, rather, as plausibly complementary extensions of it. However, I am not an expert in this area, and I don’t see the links between the main hypothesis and the subsidiary tests as being as tight as an expert might. Nevertheless, while a debate on this point may affect the shape of the logical structure of the paper, it would not, in my opinion, support an argument that there is no such structure. Nor would it challenge the conclusion that the structure is fundamentally a hypothesis-testing one. Obviously, tree diagrams similar to those in Figs. 2 and 3 could be constructed for Bai et al. (2018).

2. Independence of tests and Fisher’s Method in Bai et al. (2018)

The Reviewer also raises an important concern about the number of truly independent experiments, because the simple form of Fisher’s Method yields excessively high combined p-values if non-independent constituent p-values are used. I now reiterate this critical feature in the text, but note that when independence can be assumed, that Fisher’s test performs well (e.g., Alves and Yu, 2014, PLoS One, 9(3):91225; Poole et al., 2016, Bioinformatics, 32:i430-i436). I also argue that the issue can be dealt with by being conservative-if there is doubt about whether one test is independent of another, leave one out of the calculation. This is conservative, since including it would almost certainly increase the significance of the combined test. I agree with the Reviewer’s comment that all judgments such as those regarding independence might be “open to debate,” but argue that this does not means that judgments are invalid or worthless. Moreover, the debate about them can be illuminating. The difficulties a reader might have in deciding which experiments are truly independent remain largely unaddressed under the current system.

With regard to the specific issue of Fig. 2 in Bai et al., it is difficult to know whether the whole-brain Evans Blue measurements (2A,B) and Western Blot analyses for three different proteins at three different time points (6h, 12h, and 24h) post-surgery were done on single groups of experimental animals. The methods that the authors use, one-way ANOVA followed by a multiple-corrections comparison, should allow for data from at least one time point, say at 24h, to be used in the calculation. In any case, the authors would have to defend their reasoning for including each p-value and the p-values would have to be independent.

My goal is not to have readers become more proficient in ferreting out the logic that infuses a paper, but to persuade authors to make their reasoning plainer. If an investigator wishes to use a summarizing meta-analytic technique, then the onus is on the investigator to make the case for using it.

3. “Pre-study odds"

I have tried to correct the misapprehension that I inadvertently caused on the issue of PPV. Again, my purpose was not to defend or advocate for this parameter, but to respond to the criticisms of statisticians’ that partly rest on it. I now point out that the concept of “pre-study odds” is not unequivocally well-defined and is therefore debatable. It is particularly problematic given the statisticians’ tendency to consider every possible experimental outcome, say particular gene, to be a “hypothesis,” as opposed to the more substantive definition of scientific hypothesis as an explanation for a phenomenon. Despite the theoretical difficulty in determining what exactly “pre-study odds” means, in practical terms, it is very rare for there to be more than two or three genuine hypotheses to be at issue in a neuroscience investigation. The conclusion that hypothesis-testing projects that test only a small set of alternative hypotheses must be more dependable than open-ended searches with hundreds or thousands of conceivable hypotheses is a direct consequence of the PPV argument.

Finally, combining methods such as Fisher’s and meta-analyses and the PPV argument are entirely separate issues: combining methods could provide insightful summary analyses without PPV and vice versa, PPV calculations do not require a combination test of any kind.

4. Other methods of meta-analysis.

As the Reviewer mentions there are numerous possibilities for meta-analysis. I acknowledged as much in the original MS and, as an example, cited Winkler et al. that lists a variety of such methods. These authors discuss Fisher’s, Stouffer’s and many others as well as a variety of “non-parametric comparisons” for combining data. Both Fisher’s and Stouffer’s tests are similarly affected by correlated p-values, however, Alves and Yu (2014) demonstrate that Fisher’s Method generally outperforms Stouffer’s under these conditions. Nevertheless, no test is perfect.

The use of combination tests that the Reviewer mentions, where the “same experiment is replicated by different people using different animals,” would be a conventional context for meta-analysis. In contrast, my primary intention was not to champion any particular method, but rather to put forward the concept of using combination methods to summarize the different results of a multi-faceted test of a hypothesis.

5. Other comments

a. Calculate post-hoc statistical power of Fisher’s Method? An excellent question. According to Winkler et al., it is possible to calculate something like statistical power, but it must be done numerically and is difficult. In view of the uncertainties necessarily surrounding all of these statistical procedures, it is apparently impractical.

b. Is Fisher’s test “one-sided?” Another interesting question which is related to the previous one. Fisher’s Method tests a “global null” statistical hypothesis: namely, that none of the independent probability tests “partial nulls” that it combines are null. Fisher’s Method is an example of a “union-intersection” test that expressly includes non-identical datasets. Hence, there is not a single null “side” of a distribution. It is more accurate to think of Fisher’s test as having a multi-dimenstional “null space,” which is the intersection of all true null results (see e.g., Fig. 1 of Winkler et al. 2016) rather than a side.
